# Design and Early Use of the Nationally Implemented Healthier You National Health Service Digital Diabetes Prevention Programme: Mixed Methods Study

**DOI:** 10.2196/47436

**Published:** 2023-08-17

**Authors:** Jamie Ross, Rhiannon E Hawkes, Lisa M Miles, Sarah Cotterill, Peter Bower, Elizabeth Murray

**Affiliations:** 1 Centre for Primary Care Wolfson Institute of Population Health Barts and The London School of Medicine and Dentistry Queen Mary University of London London United Kingdom; 2 Manchester Centre for Health Psychology, Division of Psychology and Mental Health, School of Health Sciences Faculty of Biology, Medicine and Health University of Manchester Manchester United Kingdom; 3 Centre for Biostatistics Division of Population Health, Health Services Research & Primary Care, School of Health Sciences, Faculty of Biology, Medicine and Health University of Manchester Manchester United Kingdom; 4 NIHR Applied Research Collaboration Greater Manchester Centre for Primary Care and Health Services Research, Division of Population Health, Health Services Research & Primary Care, School of Health Sciences, Faculty of Biology, Medicine and Health University of Manchester Manchester United Kingdom; 5 e-health unit Department of Primary Care and Population Health, Institute of Epidemiology & Health Care University College London London United Kingdom

**Keywords:** digital health, engagement, diabetes prevention, mobile phone

## Abstract

**Background:**

The Healthier You National Health Service Digital Diabetes Prevention Programme (NHS-digital-DPP) is a 9-month digital behavior change intervention delivered by 4 independent providers that is implemented nationally across England. No studies have explored the design features included by service providers of digital diabetes prevention programs to promote engagement, and little is known about how participants of nationally implemented digital diabetes prevention programs such as this one make use of them.

**Objective:**

This study aimed to understand engagement with the NHS-digital-DPP. The specific objectives were to describe how engagement with the NHS-digital-DPP is promoted via design features and strategies and describe participants’ early engagement with the NHS-digital-DPP apps.

**Methods:**

Mixed methods were used. The qualitative study was a secondary analysis of documents detailing the NHS-digital-DPP intervention design and interviews with program developers (n=6). Data were deductively coded according to an established framework of engagement with digital health interventions. For the quantitative study, anonymous use data collected over 9 months for each provider representing participants’ first 30 days of use of the apps were obtained for participants enrolled in the NHS-digital-DPP. Use data fields were categorized into 4 intervention features (*Track*, *Learn*, *Coach Interactions*, and *Peer Support*). The amount of engagement with the intervention features was calculated for the entire cohort, and the differences between providers were explored statistically.

**Results:**

Data were available for 12,857 participants who enrolled in the NHS-digital-DPP during the data collection phase. Overall, 94.37% (12,133/12,857) of those enrolled engaged with the apps in the first 30 days. The median (IQR) number of days of use was 11 (2-25). *Track* features were engaged with the most (number of tracking events: median 46, IQR 3-22), and *Peer Support* features were the least engaged with, a median value of 0 (IQR 0-0). Differences in engagement with features were observed across providers. Qualitative findings offer explanations for the variations, including suggesting the importance of health coaches, reminders, and regular content updates to facilitate early engagement.

**Conclusions:**

Almost all participants in the NHS-digital-DPP started using the apps. Differences across providers identified by the mixed methods analysis provide the opportunity to identify features that are important for engagement with digital health interventions and could inform the design of other digital behavior change interventions.

## Introduction

### Background

Diabetes is a global health priority. The World Health Organization estimates that diabetes was the seventh leading cause of death worldwide in 2016 [1]. In the United Kingdom, approximately 3.9 million people are diagnosed with diabetes, of whom 90% are diagnosed with type 2 diabetes mellitus (T2DM) [2], and a further 5 million people are estimated to have nondiabetic hyperglycemia (raised blood glucose levels or prediabetes) in England [3]. T2DM is associated with obesity and lack of physical activity and, for many people, may be preventable through changes in diet and activity [4,5].

As such, diabetes prevention has become a public health priority. Many countries have introduced diabetes prevention programs (DPPs) that target those at the highest risk of T2DM and encourage changes in diet and physical activity. There is evidence suggesting that face-to-face, group-based DPPs can be effective in reducing the incidence of diabetes [6,7].

In England, people at high risk of developing T2DM (glycated hemoglobin of 42-47 mmol/mol [6%-6.4%] or fasting plasma glucose of 5.5-6.9 mmol/L) are offered participation by the National Health Service (NHS) in the Healthier You NHS Diabetes Prevention Programme, where they receive educational materials and in-person group sessions over 9 months to support the lifestyle changes (eg, healthier eating and increased physical activity) needed to achieve and maintain a healthy weight [8]. Despite the success of DPPs, including the Healthier You NHS Diabetes Prevention Programme [9-11], barriers such as transportation difficulties, lack of community spaces, and work or family commitments can make attendance to in-person DPPs difficult [12-14].

Digital health interventions (DHIs) have been shown to be effective in increasing physical activity, changing diets, and promoting weight loss in general populations [15,16], and there is emerging evidence suggesting that DPPs can be delivered effectively using digital technologies [17-20] and achieve results comparable with those of their in-person counterparts [17,21,22]. Digital programs delivering content (eg, physical activity and diet tracking and goal setting) through computers or smartphones while facilitating remote communication with health coaches and other participants via video calls and social forums [20,23] may also be more acceptable to some people than group-based programs and overcome some of the barriers, including time and transportation [17]. Additional drivers for the use of DHIs include the potential for scalability across large populations as well as some evidence of their cost-effectiveness [24]. However, there are considerable uncertainties around these potential advantages and little evidence yet of the effectiveness of digitally delivered DPPs in real-world populations. There are also known challenges with the wide-scale deployment of DHIs, including implementation [25,26], the digital divide and impact on health inequalities [27], and uptake and engagement by participants [28].

### Engagement With DHIs

Although DHIs can facilitate health behavior change, the extent to which they can deliver positive health outcomes depends on their successful use by users and the subsequent sustained performance of the intended health behaviors [29]. Engagement with DHIs has been defined as both an objective measure of DHI use, such as the amount, frequency, duration, and depth of the DHI accessed, and a subjective experience characterized by attention, interest, and affect [30].

Research has begun to explore intervention features that promote engagement with DHIs. Recent reviews across various health domains have reported that well-designed reminders, self-monitoring features, and embedded health professional support may promote user engagement [30-33]. However, DHI design features are not always well described, limiting understanding of how specific features may influence engagement and outcomes [34].

The relationship between engagement with digital DPPs and outcomes is not yet well defined in the literature, although there is emerging evidence indicating that greater engagement is associated with improved outcomes [18,35-37]. Furthermore, studies suggest that engagement with different features of digital DPPs may affect outcomes in different ways. For example, the study by Sepah et al [18] found that website log-ins and group participation were significantly associated with 1-year weight loss, whereas completing lessons, weighing in, and tracking steps were not. A study by Michaelides et al [35] found that the frequencies of weighing in and meals logged but not the number of group posts were significant predictors of a 6-month weight loss. A pilot study of the Healthier You NHS Digital Diabetes Prevention Programme (NHS-digital-DPP) found that those participants who had access to peer support as part of the intervention had greater weight loss than those without [17].

Studies of the NHS-digital-DPP have described the features of the interventions [38,39], people’s attitudes toward use [28], and the features that people engage with over the 9-month program [40]. However, there has yet to be any research exploring the way in which service providers have designed the NHS-digital-DPP to promote participant engagement or describing early use.

Researchers have expressed concern about how little detail is provided in relation to the underlying principles of the development and design processes of health apps [41-43] and subsequent expectations in terms of use and engagement for these interventions [42]. Understanding whether users’ actual engagement aligns with the design expectations of the developers is a critical step toward enhancing uptake and engagement with interventions [43]. This may be particularly important for apps such as those that comprise the NHS-digital-DPP, which are made available to participants for *real-world* use. Previous studies have suggested that, in contrast to engagement and use within research settings, participants who make use of DHIs in the real world do not engage as much or as intended by intervention developers [44]. A recent systematic comparison of published reports and real-world use of the same DHIs reported that users who participated in trials had 4 times higher eHealth program use than real-world users of the same programs [45].

### Goals of This Study

This study adds to the digital DPP translation literature in 2 ways. First, it seeks to understand how the NHS-digital-DPP has been designed to promote participant engagement. Second, it presents data on early engagement with the apps that comprise the NHS-digital-DPP, presenting one of the few examples of an analysis of routinely collected use data from this population.

The focus of this study was on early engagement, encapsulating a period that may represent participants’ initiation and early sustained use [46,47]. Previous research indicates that the number of people who engage and the amount of engagement are higher at the start of intervention use compared with later [36,48]. Furthermore, previous studies on weight loss programs have shown initial weight loss (first month) to be high, followed by a slowing over time [49], suggesting that this is an important period to study.

Providers of the NHS-digital-DPP record participants’ data over the 9-month duration of the program in 30-day intervals called *engagement periods* as required by NHS commissioners [8,50]. The use data analysis presented here represents the first 30 days of app engagement for all participants who enrolled with 3 of the 4 providers of the NHS-digital-DPP over a 9-month period. Another study by this group reports on a longitudinal analysis of use data for a cohort of participants [40].

### Aim

This study aimed to explore engagement with the NHS-digital-DPP. The specific research questions (RQs) were as follows: How have the providers of the NHS-digital-DPP designed their interventions to promote participant engagement? (RQ 1); and How do participants engage with the apps that comprise the nationally implemented NHS-digital-DPP during the first 30 days of enrollment? (RQ 2).

## Methods

### Design

We used a mixed methods study design with qualitative and quantitative methods [51,52]. RQ 1 was addressed via the qualitative component comprising semistructured interviews and an analysis of specification documents from the 4 providers of the NHS-digital-DPP. RQ 2 was addressed via the quantitative component, an analysis of anonymized participant use data collected from the apps of 3 of the 4 providers of the NHS-digital-DPP.

Qualitative data were collected to allow for an in-depth understanding of the way in which the NHS-digital-DPP was designed to promote participant engagement (RQ 1). Quantitative data were collected to capture objective measures of app engagement by participants (RQ 2). Quantitative and qualitative data were collected independently during the same period. As each method addressed a separate objective, data were kept separate during analysis and synthesized at the point of data interpretation.

### NHS-Digital-DPP Intervention

The NHS-digital-DPP was delivered by 4 independent providers commissioned by NHS England [39]. To claim payments, providers must be able to demonstrate that users had engaged. Engagement was defined by the NHS as a minimum of 2 episodes of active engagement within at least 1 of 6 categories of engagement in each 30-day period: communication with a health coach, accessing educational content, logging information against goals, peer support forums, use of interactive tools, and time spent in the app [22]. Although based on a common service specification [8], providers vary in terms of their provision of materials, inclusion of wearables (eg, accelerometers and wireless weighing scales), the amount of human support provided (ranging from a brief onboarding phone call to weekly coaching phone calls), the delivery platform (smartphone app or website), and the amount and format of educational materials (eg, websites and emails) [39]. Table 1 provides a summary of provider features that have been described in detail by this team previously [39,40], and further descriptions are provided in Multimedia Appendix 1 [30].

**Table 1:** Summary of features of the National Health Service Digital Diabetes Prevention Programme.

	Provider A	Provider B	Provider C	Provider D
Delivery mode^a^	App and desktop format	App and desktop format	App	App and desktop format
Materials provided^b^	Web-based learning platform (also available on the app) with 42 lessons comprising articles that included text, images, videos, podcasts, and links to external websites Content unlocked weekly throughout 9 months	Program handbook Weekly web-based educational articles delivered via a range of channels, including text and video	Tailored educational articles and videos sent from health coach to service users via message Content sent weekly (months 1-3), biweekly (months 4-6), and monthly (months 7-9) over the 9-month program	Web-based learning platform (also available on the app) comprising articles that included text, images, videos, podcasts, and links to external websites Content unlocked weekly during the first 3 months of the program 7 optional “Sustain” courses through months 4-7 of the program with more in-depth information on education topics Program handbook, recipe book, wireless scales, and activity tracker
Professional input	Monthly telephone call with health coach to discuss progress and review goals Health coaches send messages to users and provide feedback on behaviors and outcomes tracked in the app	Optional access to health coaches via chat function	Initial 45-minute video call with health coach to discuss program and set goals Regular messages from health coach throughout the 9-month program, including receiving educational content and feedback on behaviors and outcomes tracked in the app; health coaches check in with users weekly, then biweekly, and then monthly	Support from health coach via the group chat during the first 3 months of the program; one-to-one messaging also available during the first 3 months of the program
Peer support	Optional closed group chats moderated by health coach^c^	Open group discussion forum available on the app throughout the 9-month program	Open group discussion forum available on the app throughout the 9-month program	Users allocated to a closed group chat (10-15 people; available on the app) moderated by a health coach for the first 3 months of the program; closed group is available through months 4-9 without the health coach moderation Open group discussion forum available on the app in months 4-9

^a^All providers had goal setting and self-monitoring functions on their apps.

^b^All providers covered topics such as dietary fiber, alcohol, physical activity, managing stress, sleep, and managing social events.

^c^Not all participants enrolled with this provider opted for the pathway that provided access to peer messaging.

Individuals eligible for the NHS-digital-DPP were adults aged ≥18 years diagnosed with nondiabetic hyperglycemia. This is defined as having at least one glycated hemoglobin reading of 42 to 47 mmol/mol or at least one fasting blood glucose reading of 5.5 to 6.9 mmol/L in the 24 months before referral. People already diagnosed with diabetes and pregnant women were not eligible for the program. Individuals were referred to the program either via their general practice if their records indicated that they had nondiabetic hyperglycemia or via a web-based risk assessment tool [53], which included questions on age, gender, ethnicity, waist circumference, and body weight. If the questionnaire deemed individuals to be at medium or high risk, they were eligible for self-referral to the program. Those who took up the referral were enrolled in 1 of the 4 service providers’ digital programs depending on which provider was commissioned to deliver the digital service in their local geographical area across England in 2019 to 2021.

### Data Collection

#### Provider Interviews and Document Review

Intervention design documents were collected, and interviews with program developers from each of the 4 providers were conducted. Full methods for data collection of the documents and qualitative interviews are reported in an earlier study [54], and a summary is provided in this section.

The following documents describing the design of the NHS-digital-DPP were obtained from the digital providers: (1) providers’ framework response bids describing digital providers’ proposed service delivery submitted to NHS England during service procurement, 1 per provider (n=4), and (2) additional documentation supplied by 3 providers that detailed further information about the planned behavior change content designed in their digital programs.

Interviews were conducted with program developers employed by each of the 4 providers. Providers were private sector organizations that each secured contracts to deliver the NHS-digital-DPP in 2019 to 2021, and program developers were employed by each in the design and development of the NHS-digital-DPP.

Sampling aimed to include professionals from different backgrounds who had different roles in program development to gather a range of views and provide a comprehensive understanding of the processes involved in the design and development of each NHS-digital-DPP intervention. Participants were recruited for the study via email through digital provider leads. Sampling was initially convenient, with health coaches who were interested in taking part contacting the research team, and snowball sampling identified further participants. Full recorded verbal consent was obtained from each participant before commencing the interview, including for further analysis by members of the research team.

The questions addressed the strategies and intervention features used by the providers to promote participant engagement as well as the expectations that providers had regarding how individuals engage with the apps. The interviews were conducted via a video call platform (Zoom; Zoom Video Communications). Verbal consent was obtained from each participant via an encrypted audio recorder.

Interviews were conducted with 6 professionals involved in the design and delivery of the NHS-digital-DPP. Interviews with 2 professionals took place for providers A and C, and interviews with 1 professional took place for providers B and D. Participants were 67% (4/6) female and 33% (2/6) male and had backgrounds in dietetics or nutrition (3/6, 50%), exercise science (2/6, 33%), biomedical science (1/6, 17%), and as a previous service user (1/6, 17%). Their roles at the time of the interview included clinical lead (1/6, 17%), service delivery lead for diabetes prevention (1/6, 17%), head of health solutions (1/6, 17%), clinical services manager (1/6, 17%), head of coaching programs (1/6, 17%), and manager of NHS contracts (1/6, 17%).

The interviews were transcribed verbatim.

#### Use Data

A sample of routinely collected anonymous app use data for participants enrolled in the NHS-digital-DPP during a period between December 2020 and February 2022 was obtained from 75% (3/4) of the providers. One provider was unable to provide data.

An opt-out consent statement was present in each provider’s terms and conditions, and privacy policies before participants signed up to the apps. Data were encrypted, password protected, and sent via email to the research team. All data were stored on a secure university server only accessible to the research team.

The analysis of the use data explored how participants initially engaged with the apps. Initial engagement was defined as the first 30 days of use following registration. The first 30 days of data were requested from the 3 providers for every new registrant. Data were collected for 9 months for each provider, but the dates of data collection differed for each provider.

In total, providers collected data from 12,857 participants. Provider A collected data from 9821 (76.39% of total sample 12,857 participants) participants between May 2021 and February 2022, provider C collected data from 1087 (8.45%) participants between December 2020 and August 2021, and provider D collected data from 1949 (15.16%) participants between December 2020 and August 2021. These samples reflect the total sample enrolled to the provider’s programs during this time period.

### Data Analysis

#### Qualitative Analysis

Documents and interview transcripts were treated as one data set and analyzed using NVivo (version 12; QSR International) [55] software. JR deductively coded all data using a *DHI engagement framework* of direct and indirect influences on engagement with digital behavior change interventions that was developed from a critical interpretive synthesis of 117 studies [30] (see Multimedia Appendix 1 [30] for a description of this model). All framework concepts and their constituent attributes that related to features of the interventions themselves (eg, as opposed to attributes of participants) were assigned as a priori codes (see Table 2 for a list of the constituent attributes of the concepts *Delivery* and *Content*). The framework’s 2 overarching concepts of *Delivery* and *Content* formed the higher-level codes, whereas their constituent attributes (eg, for *Delivery*, these included “novelty” and “personalization,” and for *Content*, these included “social support features” and “reminders”) were lower-level codes. All data were read line by line, and data relevant to engagement with the NHS-digital-DPP were assigned to one of these a priori codes. During coding, an additional factor was identified from these data that was not encompassed by the engagement framework. This was labeled as the behavior change technique “problem solving,” defined by Michie et al [56] as “Analyse, or prompt the person to analyse, factors influencing the behaviour and generate or select strategies that include overcoming barriers and/or increasing facilitators.” In this data set, it related to instances of participants being supported to understand why their engagement with the apps was low and identify ways to overcome this, such as technical support.

**Table 2:** Summary of engagement features described in provider interviews.

DHI^a^ engagement framework factors		Provider A	Provider B	Provider C	Provider D
**Content**					
	Feedback	✓^b^			
	Goal setting	✓	✓	✓	
	Reminders	✓	✓	✓	✓
	Rewards		✓		
	Self-monitoring		✓		✓
	Social support features		✓	✓	✓
	Problem-solving^c^	✓		✓	
**Delivery**					
	Esthetics and design				✓
	Challenge^d^				
	Complexity^d^				
	Control features (sequentiality of content delivery)	✓	✓	✓	✓
	Credibility features			✓	✓
	Ease of use	✓	✓	✓	✓
	Familiarity^d^				
	Guidance	✓	✓	✓	✓
	Interactivity		✓		
	Message tone			✓	
	Mode of delivery	✓	✓	✓	✓
	Novelty		✓		✓
	Narrative^d^				
	Personalization	✓	✓	✓	
	Professional support features	✓	✓	✓	✓

^a^DHI: digital health intervention.

^b^Factor present in the data for the provider.

^c^Not included in the DHI engagement framework but identified from the data.

^d^Factor absent from all providers’ data.

#### Statistical Analysis

App use data detailing what participants had engaged with and when were provided by 3 NHS-digital-DPP providers in monthly Microsoft Excel (Microsoft Corp) spreadsheets. Data for each provider were combined to make a total data set per provider and were then cleaned to remove duplicates, blank cases, and cases not related to the first 30 days of use. The data were then transferred to SPSS (IBM Corp) [57] for analysis.

In total, 2 variables were available for all 3 providers to describe an individual’s *amount* of engagement: time spent and number of days using the apps. To explore *how* the apps were engaged with, the types of engagement with the apps were categorized as *Track*, *Learn*, *Peer Support*, or *Coach* in line with the NHS categories of engagement [22]. *Track* engagement included setting or amending a goal or self-monitoring of behavior (eg, diet or physical activity) or outcome (eg, weight). *Learn* represented unique articles accessed by participants (providers A and D) or sent to participants (provider C). *Peer Support* engagement included group posts, comments, or “likes” posted in peer discussion forums (providers C and D) and private messages sent to peers (providers A and D). *Coach* engagement included messages or phone calls with health coaches (phone calls and messages were recorded separately by providers; Table 3).

Descriptive analyses were carried out to describe the overall engagement with the apps and how participants engaged with them. All variables were assessed through Kolmogorov-Smirnov tests of normality as non–normally distributed; as such, median values and IQRs are presented. To compare engagement across providers, Kruskal-Wallis nonparametric tests comparing independent samples were conducted on all variables, and Mann-Whitney *U* tests were used to assess differences between providers.

**Table 3:** Summary of engagement measures included in use data analysis across providers.

		Provider A	Provider C	Provider D
Amount of engagement		Number of minutes spent on the app Number of days with recorded interactions	Number of minutes spent on the app Number of days users opened the app	Number of minutes spent on the app Number of days with recorded interactions
**Type of engagement**				
	Track	Number of times behaviors were self-monitored Number of times outcomes were self-monitored Number of goals set or amended (behavioral or outcome)	Number of times behaviors were self-monitored Number of times outcomes were self-monitored Number of goals set or amended (behavioral or outcome)	Number of times behaviors were self-monitored Number of times outcomes were self-monitored Number of goals set or amended (behavioral or outcome)
	Learn	Number of times educational articles were accessed	Number of educational materials sent to participants	Number of times educational articles were accessed
	Peer support	Number of sent peer messages (message sent by the participant in the group chat)^a^	Number of group posts (open group discussion forums) Number of comments on posts (open group discussion forums) Number of likes on posts (open group discussion forums)	Number of sent peer messages (in closed peer group chat moderated by a health coach) Number of group posts (open group discussion forums) Number of comments on posts (open group discussion forums) Number of likes on posts (open group discussion forums)
	Coach interactions	Number of calls with health coach Number of messages with health coach	Number of calls with health coach Number of messages with health coach	Number of messages with health coach

^a^Not all participants enrolled with this provider opted for the pathway that provided access to peer messaging.

### Ethics Approval

The wider program of research that this study is a part of was reviewed and approved by the North West–Greater Manchester East NHS Research Ethics Committee (reference 17/NW/0426; August 1, 2017).

### Participation

No payments were made for participation. All presented data were anonymized.

## Results

### How Has the NHS-Digital-DPP Been Designed to Promote Participant Engagement?

#### Targets for Engagement

Generally, providers had pragmatic expectations of how individuals would engage with their programs, acknowledging that the amount and patterns of engagement would vary across participants:

You get different personality profiles, people who have different engagement styles.

Provider C

It’s so scattered. Some people will engage—you know, barely engage with some parts of the intervention and occasionally engage with others...And then you have the other side of the spectrum I would say...where they will engage for hours a day.

Provider D

However, for all providers, there was agreement that, for someone to be viewed as “engaged” with the apps, this had to be reflected in some amount of daily use:

For our engaged people, we are talking about multiple uses of the app every day.

Provider B

There’s daily conversations in—for the first twelve weeks the engagement level of the intervention is meant to be every day.

Provider D

#### Engagement Features

The app features that providers described as promoting engagement are summarized in Table 2. Several features were described by all 4 providers: reminders, control features, ease of use, guidance, mode of delivery, and professional support features. Some features of the DHI engagement framework [30] were not reported in this data set; these were challenge, complexity, familiarity, and narrative. Providers reported strategies to engage participants at 3 time points: initiation, sustained engagement, and the point of disengagement.

#### Initiation

The first contact that individuals had with the NHS-digital-DPP was viewed as a critical time to establish and promote engagement. “Initial assessments” or “onboarding” sessions were used by all providers, which comprised an initial phone or video call with a professional associated with the digital service. There were common strategies used during these initial conversations, which were designed to engage participants with the apps. Providers tried to minimize the time between someone registering interest and having an onboarding call to capture people when they were perceived to be most motivated to join.

Providers spoke about trying to make it as easy as possible for participants to engage with the digital program, using different delivery modes and providing technical assistance when needed:

We’re very, very clear about making it as easy as we possibly can for people to engage digitally. Um, so we have a welcome pack that we sent out. We sent them some instructions on how to get on board obviously. How to access the digital app. So full details is actually given. Um, and that’s served up via a video, so we also send pdf materials through in terms of an overview of our approach, a pdf of our diabetes work but which includes lots of curriculum content as well, and we send them everything that they need to get their digital account set up.

Provider B

Providers spoke about the importance of having a human element to the digital DPP, especially initially, to build commitment to engaging with the programs:

And I think it [having a health coach] really humanises the programme as well, which could be looked at as potentially quite cold if it’s perceived as tech only. So that’s really nice, and that helps to break down barriers from my perspective, or at least my opinion when it comes to signing up to a programme like this.

Provider C

For one provider (provider C), this human element was a core component of onboarding participants to the service, whereas for others, this contact was reserved for those who were having difficulties getting started (provider A):

We do have a self-led onboarding for patients, so it’s sort of more so those patients that do need a bit more, um, nudging that we generally tend to interview.

Provider A

Provider C described these onboarding calls as an opportunity to secure participant commitment to engaging with the service by making a verbal commitment to the coach:

The patient hopefully in most cases, makes the agreement and this could happen through text or through video message. The patient will make the commitment to registering accurate information against the goals. Logging into the app regularly. Being truthful about the information. Being open and honest about how they’re finding things. Commitment to the programme length which is nine months in this case, you know it’s a big programme, most people don’t expect that. And also again that’s set in place that both the patient and the coach can always refer back to.

Provider C

All 4 providers emphasized the importance of ease of use at this critical initiation stage and spoke of the ways in which they had streamlined processes to achieve this, for example, by removing unnecessary steps for participants and making all processes achievable using the same technology to reduce burden:

But what we do is we’re very, very clear about making it as easy as we possibly can for people to engage digitally.

Provider B

Providing guidance at this stage was perceived as crucial to support participants in gaining access to the apps and being able to use them. Guidance was mentioned by all providers and included instruction materials; calls with staff to demonstrate navigation around the apps, highlight key features, and troubleshoot; self-help videos; and support networks:

We do have things in place in terms of supporting patients to get to grips with the app. So another part of that initial consultation is to support patients in navigating around the app. And we’ve obviously got help videos, we’ve got support networks that are available within the app, and there’s spaces where patients can ask questions, support each other.

Provider C

#### Sustained Engagement

Once participants were onboarded to the apps, providers spoke of many strategies that were used to maintain and promote engagement.

There were mentions of specific behavior change techniques that were used to promote engagement with the apps, including feedback, goal setting, and self-monitoring, which were operationalized via tools or features of the apps that promoted regular participant engagement. One provider (provider C) suggested that the range of goals that participants were able to set was engaging and explained that they provided multiple options to appeal to as many people as possible. Provider D set the expectation with participants at the initial assessment that they should self-monitor their behaviors on a daily basis, encouraging daily use to record them:

People are encouraged to self-monitor daily changes in their steps, sleep, weight and so it is, kind of, designed around daily engagement.

Provider D

Prompts and reminders were described by all providers as techniques used to promote engagement. Prompts were used to “nudge” (provider A) participants to complete outstanding tasks, prompt participants to engage more regularly, and remind participants to engage with their coaches. The delivery of prompts and reminders varied across providers, with some prompting all participants on a regular basis (eg, fortnightly in the case of provider B) to engage with tools and curriculum, whereas others targeted prompts at those who were not engaging at perceived optimum levels:

Essentially if a patient completes all of their goals for a day, they get a congratulatory message. If they don’t, they get a reminder to go and complete their goals. Um, that’s the bread and butter simple one notification a day.

Provider C

One provider (provider C) described a fine balance when sending reminders to participants between promoting engagement and risking disengagement because of participants being annoyed by regular reminders:

Getting right in a digital world is important. Again, trying to find a way to engage people and not “p” them off, it’s quite difficult.

Provider C

Other strategies to maintain and promote engagement included problem-solving with participants (provider A), which helped identify whether there were any technical barriers to use. One provider (provider B) described using rewards as a strategy to promote engagement, and another provider sent congratulatory messages to those who interacted with the apps (provider C):

So for example accessing various parts of the app, um, visiting particular pieces of content like recipes. So the more active you are, within the digital space, the more points you will win.

Provider B

Providers spoke about the role of social support features, including discussion forums and messaging, in engaging participants. It was acknowledged that, although peer support may build participant accountability and engagement for some, these support features were demotivating and frustrating for others. Provider D, who offered closed group chats (such as WhatsApp) and discussion forums (such as Facebook), talked about the importance of matching participants in the closed groups based on certain characteristics (such as age), which has been shown to facilitate discussions. Provider C referred to ongoing efforts to try to maximize participation in their discussion forum, including analyzing what the optimum group sizes should be and what interactive functions there should be:

It’s not quite social media I don’t think but, um, that’s obviously a very powerful driver for a lot of people who are very social. And want to—who do build relationships with people and they want to see how they’re getting on, and they want to support them. And they end up forming bonds and relationships and that’s what brings them back to engage in the application.

Provider D

The content delivery strategies used by providers were deemed important for engagement. A sequential delivery of educational materials such as articles was used by providers A, C, and D. For example, provider A released new content to participants every 30 days with accompanying reminders, whereas provider D released new content on a daily basis. Provider C released new content at different time points depending on a participant’s interaction with the materials. These 3 providers described this drip feeding of content as better at promoting engagement with the apps and providing novelty than participants being able to access all content in one go:

We do have content that sort of is made available as users progress, so, um, it sort of means that we are more likely to get people to come back and continue to use it over a longer period of time, versus logging on once, reading everything and then just never going back to it again.

Provider A

In addition to the timing of content delivery, a range of delivery modes was also described as important for engaging as many participants as possible, taking into consideration different learning styles and preferences. Provider A talked about using a range of approaches to contact participants, including email, phone calls, and SMS text messages. In total, 50% (2/4) of the providers described establishing participant preferences for the content delivery mode during the initial assessments:

In terms of digital engagement, we look at how best to engage people in terms of engagement profiles with messages, videos, features, one-to-one, peer-to-peer, how should we send notifications and nudges. You know, when you annoy people versus when you engage people.

Provider C

Personalization was also built into the apps in terms of people’s preferences for engaging with their coaches, the type of plan they wanted to follow (provider B), the types of information they wanted to receive (provider A), tailoring of notifications (provider C), and the ability to select a health coach (provider C). These preferences were established via the coaches through interactive tools and gamification features.

For 75% (3/4) of the providers (A, C, and D), a core part of promoting engagement centered on input from health coaches. This input was described as enhancing accountability (providers C and D) and providing motivation to maintain engagement (providers C and D). For these providers, health coaches were responsible for proactively engaging participants to use the apps, for example, by delivering content to participants, posting in group chats, and sending messages:

And, you know, having someone there who responds to you is an incredibly valuable thing to help people change their behaviour and continue engaging with the intervention. And build that accountability which is the basis of interactions between patients and healthcare professionals really.

Provider D

Provider C suggested that the presence of a health coach made it harder for people to disengage from the app compared with a purely digital offering because of the connection established between participants and health coaches:

So they’re not engaging with an app in our service, they’re engaging with a real person who they trust. And in the same way if you don’t delete people from your phone once you’ve got connection and your contact, most people are quite happy that that’s a connection and a contact. That’s the same way with us, whereas if it’s just an app, you know, people delete apps all the time, if it’s a game or if it’s a business app they might have used, it’s an app, it’s just an app, it’s just a piece of software.

Provider C

#### Disengagement

Disengagement from the apps was anticipated by providers. Several reasons for participants to disengage were given, including participants making good progress and, thus, not wanting to engage as much; feeling confident with the information they were being provided with and, thus, feeling less need to engage; or experiencing setbacks with progress and, thus, dropping off the intervention:

You know, people can lose a significant amount [of weight] in the first twelve weeks, and, you know, why would they want to engage for another six months, if they’re happy with what’s happened.

Provider D

Despite this, all providers had protocols in place to address disengagement. Disengagement was reported to take several forms and occur at different stages in the participant journey, for example, not engaging with the onboarding appointments, engagement dropping off over time, and someone ceasing all engagement.

Participants who were not engaging optimally (defined by providers) were automatically identified by the software, and the provider teams were alerted. This identification of participants at risk of disengaging happened at regular intervals; for example, provider B ran monthly “sweeps” for codes that had not been activated (needed to register), and provider C ran software at 14, 21, and 28 days to identify participants with low engagement:

The...system automatically identifies participants with lower engagement than the group average and therefore at higher risk of disengaging. The Coach is automatically notified and contacts the participant via telephone to discuss reasons for lower activity on the website/App and address any issues/concerns to maintain retention.

Provider D

The strategies in place to address disengagement centered on attempting to contact participants to prompt, remind, or problem solve. Using a range of contact methods was described as important, especially in cases where disengagement was a result of technical problems with the apps. Contact was attempted either by sending out automated communications, such as SMS text messages and emails, or via calls or messages from the health coaches. Providers collected data on participants’ reasons for disengaging:

On a monthly basis if we see that people are not engaging either in a primary or secondary engagement in the way that we’d like them to, then we start sending them follow-ups and that’s either—it’s not by letter, clearly they’re a digital user, whether it’s by email or text message. And we have been using some telephone calls.

Provider A

A tension between providers’ understanding of how people interact with their app and the NHS engagement framework was evident for instances of disengagement. Providers explained that they expected participants to reduce or cease engagement, but because they were adhering to the NHS engagement framework for payment purposes, they had to keep pushing people to engage:

This whole engagement structure that NHS England have put in place, is very much around us constantly having to try and—“you’re not engaging, please engage.” Even if you’ve lost ten kilos and then we get a message back saying, “Why do I need to? I feel you guys have done an amazing job. I’m completely happy with the support I’ve had and I’m really confident to go it alone.”

Provider D

### How Do Users Engage With the NHS-Digital-DPP During the First 30 Days?

#### Use Data Analysis

**Figure 1:**
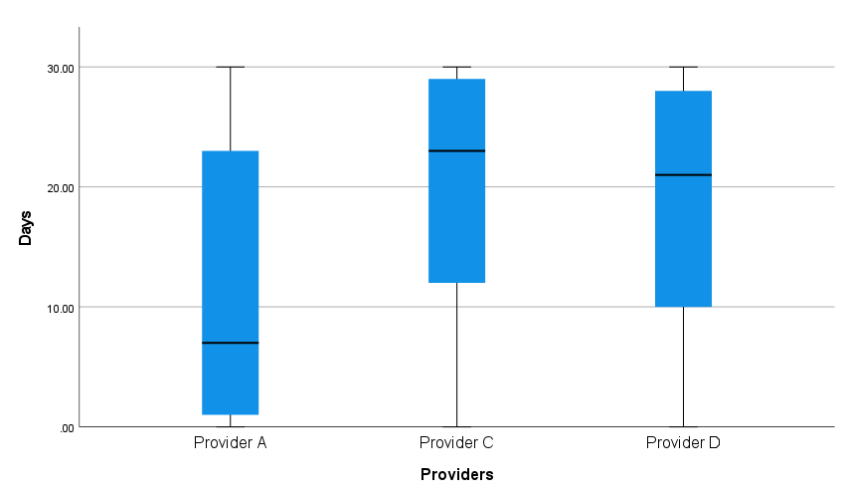
Amount (days) of use of the National Health Service Digital Diabetes Prevention Programme apps.

**Figure 2:**
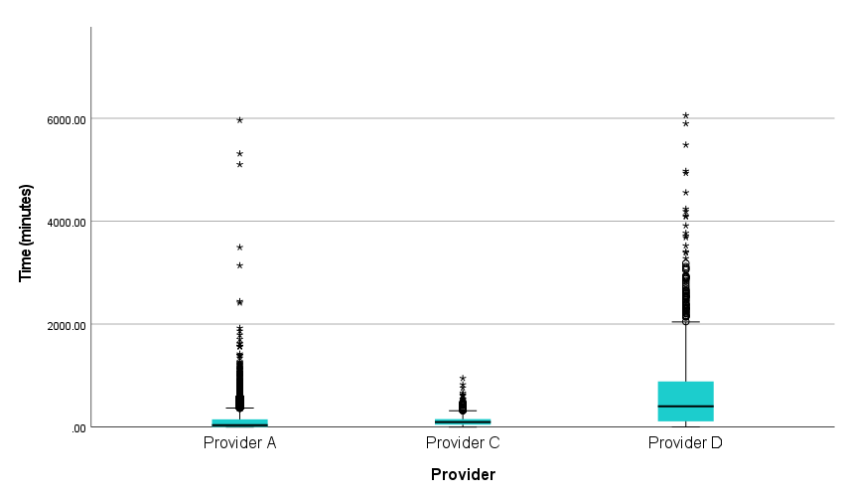
Amount (time) of use of the National Health Service Digital Diabetes Prevention Programme apps.

Data were analyzed for 12,857 participants. Each provider supplied data representing the first 30 days of engagement for all participants enrolled in the apps during the 9-month study period. Table 4 presents the median number of interactions by provider and the results of the nonparametric tests comparing interactions across providers. Table 5 presents the number and proportion of participants who engaged with each feature of the programs. These findings are described in the following sections. Figures 1 and 2 show the amount of engagement of participants and interactions with the intervention features.

**Table 4:** Recorded engagement by provider (N=12,857).

		All providers	Provider A (n=9821)	Provider C (n=1087)	Provider D (n=1949)	Kruskal-Wallis chi-square test (*df*)		*P* value	
**Time (min)^a^**							2089 (2)		<.001
	Interaction, n	2,459,779	1,109,858	129,381	1,220,540				
	Median (IQR)	60 (4-204)	35 (1-148)	95 (45-155)	404 (109-887)				
**Days^b^**							1143 (2)		<.001
	Interaction, n	170,549	112,582	22,497	36,295				
	Median (IQR)	11 (2-25)	7 (1-23)	23 (12-29)	21 (10-28)				
**Track^c^**							5 (2)		.10
	Interaction, n	1,233,380	1,000,317	64,144	168,919				
	Median (IQR)	46 (3-22)	45 (2-128)	52 (27-81)	45 (4-122)				
**Learn^d,e^**							734 (2)		<.001
	Interaction, n	351,324	206,222	5368	139,734				
	Median (IQR)	7 (1-32)	6 (1-29)	4 (0-9)	28 (2-90)				
**Coach^f^**							3373 (2)		<.001
	Interaction, n	77,985	26,876	24,668	26,441				
	Median (IQR)	1 (0-7)	1 (0-3)	20 (16-26)	6 (0-18)				
**Peer support^g^**							4337 (2)		<.001
	Interaction, n	24,567	2067	2954	19,546				
	Median (IQR)	0 (0-0)	0 (0-0)	0 (0-0)	2 (0-12)				
**All engagements^h^**							71 (2)		<.001
	Interaction, n	1,687,319	1,235,482	97,134	354,640				
	Median (IQR)	75 (13-170)	68 (10-167)	81 (51-118)	95 (15-254)				

^a^Provider A versus provider C: *P*<.001; provider A versus provider D: *P*<.001; provider C versus provider D: *P*<.001.

^b^Provider A versus provider C: *P*<.001; provider A versus provider D: *P*<.001; provider C versus provider D: *P*<.001.

^c^Provider A versus provider C: *P*<.001; provider A versus provider D: *P*=.10; provider C versus provider D: *P*>.99.

^d^Provider A versus provider C: *P*<.001; provider A versus provider D: *P*<.001; provider C versus provider D: *P*<.001.

^e^The comparison across providers for “Learn” should be interpreted with caution because of the different measures that constituted this variable across providers. Providers A and D measured their educational content with variables that reflected how many times participants accessed educational materials, whereas provider C measured how many times educational content was sent to participants.

^f^Provider A versus provider C: *P*<.001; provider A versus provider D: *P*<.001; provider C versus provider D: *P*<.001.

^g^Provider A versus provider C: *P*<.001; provider A versus provider D: *P*<.001; provider C versus provider D: *P*<.001.

^h^Provider A versus provider C: *P*<.001; provider A versus provider D: *P*<.001; provider C versus provider D: *P*<.001.

**Table 5:** Number and proportion of participants who engaged with each feature of the programs (N=12,857).

Engagement^a^	Provider A (n=9821), n (%)	Provider C (n=1087), n (%)	Provider D (n=1949), n (%)	All providers, n (%)
Days with engagement recorded	7425 (75.6)	1070 (98.43)	1948 (99.95)	10,444 (81.2)
Daily activity	893 (9.09)	201 (18.49)	335 (17.19)	1513 (11.77)
Track	8124 (82.72)	1062 (97.7)	1601 (82.14)	10,788 (83.91)
Learn^b^	7710 (78.51)	607 (55.84)	1607 (82.45)	9925 (77.2)
Coach	5439 (55.38)	1087 (100)	1451 (74.45)	7978 (62.05)
Peer	478 (4.87)	118 (10.86)	1166 (59.83)	1763 (13.71)
Any engagement during the first 30 days	9302 (94.72)	1087 (100)^c^	1744 (89.48)	12,133 (94.37)

^a^These data relate to participant engagement with the *app* components of the programs. As shown in Table 1, a total of 3 provider programs could be accessed via a desktop version of the interventions, and engagement with these platforms was not part of this analysis.

^b^The comparison across providers for “Learn” should be interpreted with caution because of the different measures that constituted this variable across providers. Providers A and D measured their educational content with variables that reflected how many times participants *accessed* educational materials, whereas provider C measured how many times educational content was *sent* to participants.

^c^This is a higher proportion than those with recorded activity (days) as any engagement included coach calls that users may not have had to open the app to engage with.

#### Overall Measures of Engagement

##### Time Spent Using the Apps

As shown in Table 4, participants from provider D spent significantly more time engaging with the app, with a median of 404 (IQR 109-887) minutes (6.7 h), compared with those from provider A, a median of 35 (IQR 1-148) minutes and provider C (median 95, IQR 45-155 min). Differences between the 3 were statistically significant (N=12,857; χ^2^_2_=2089, *P*<.001).

##### Number of Days of Recorded App Use

Across all providers, participants engaged for a median of 11 (IQR 2-25) days with the apps, with 81.23% (10,444/12,857) of participants having some recorded engagement on one or more days. There were significant differences in overall engagement (days) with the apps by provider (N=12,857; χ^2^_2_=1143, *P*<.001). Participants from provider C used the app for a median of 23 (IQR 12-29) days compared with 7 (IQR 1-23) days for those from provider A and 21 (IQR 10-28) days for those from provider D. Differences among the 3 providers were all significant.

#### Engagement With App Features

##### Overview

**Figure 3:**
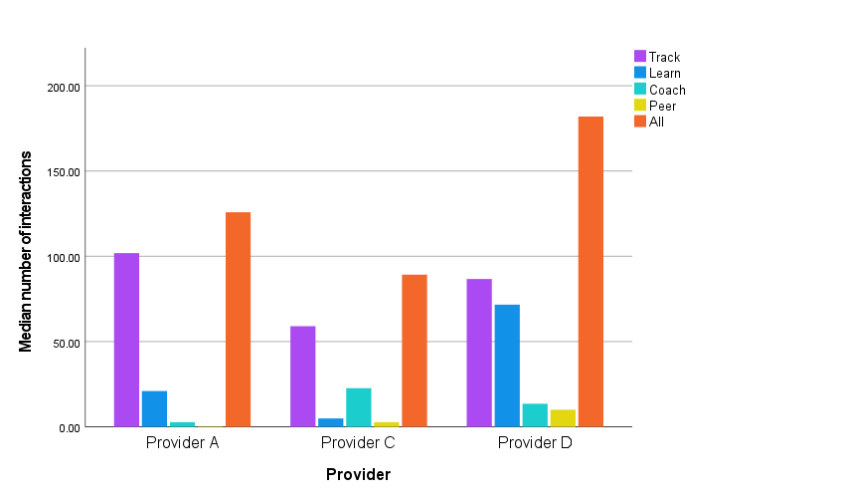
Types of engagement with the National Health Service Digital Diabetes Prevention Programme.

Overall, participants had a median of 75 (IQR 13-170) engagements with the apps, and 94.37% (12,133/12,857) of all participants engaged in some way with the apps. There were significant differences between providers in terms of overall app engagement (N=12,857; χ^2^_2_=71, *P*<.001). Participants from provider D had more engagement in the first 30 days (median 95, IQR 15-254) than those from provider A (median 68, IQR 10-167) and provider C (median 81, IQR 51-118); all differences were significant. All participants (1087/1087, 100%) from provider C had a record of engaging with the app compared with 94.72% (9302/9821) of participants from provider A and 89.48% (1744/1949) of participants from provider D. Figure 3 shows engagement with features of the NHS-digital-DPP apps across providers.

##### Track

Tracking features were the most commonly engaged with components of the apps, with 83.91% (10,788/12,857) of all participants engaging with a tracking feature and a median of 46 (IQR 3-22) tracking interactions per person. There were no statistically significant differences between providers (N=12,857; χ^2^_2_=4.9, *P*=.09). Tracking was engaged with by a high proportion of participants across providers, with 82.71% (8124/9821), 97.7% (1062/1087), and 82.36% (1601/1944) of participants from providers A, C, and D, respectively, engaging in tracking activities (goal setting or self-monitoring of behaviors and outcomes).

##### Learn

Overall, 77.2% (9925/12,857) of all participants had either accessed (providers A and D) or been sent (provider C) educational material via the apps, with a median across providers of 7 engagements per person (IQR 31). Significant differences were observed across providers for *Learn* engagement (N=12,857; χ^2^_2_=734, *P*<.001). Participants from provider D accessed a median of 28 (IQR 2-90) educational materials, and provider A participants accessed a median of 6 (IQR 1-29). Provider D had a higher proportion of participants accessing the materials (1607/1944, 82.66% compared with 7710/9821, 78.51%). Health coaches from provider C sent 55.84% (607/1087) of participants a median of 4 (IQR 0-9) articles.

##### Coach Interactions

Overall, 62.05% (7978/12,857) of participants had engaged with a health coach via the apps on at least one occasion in the form of phone calls, messages, or both. A median of 1 (IQR 0-7) engagement was recorded with coaches for all participants. There were statistically significant differences across providers (N=12,857; χ^2^_2_=3373, *P*<.001) for engagement with coaches. Participants from provider C had a median of 20 (IQR 16-26) engagements compared with those from provider A (median 1, IQR 0-7) and provider D (median 6, IQR 0-18); all differences were statistically significant. All participants (1087/1087, 100%) from provider C had engaged with coaches within the first 30 days of enrolling in the program compared with only 55.38% (5439/9821) of participants from provider A and 74.64% (1451/1944) of participants from provider D.

##### Peer Support Features

Overall, app peer support features were rarely used by participants from any provider, with only 13.71% (1763/12,857) of participants having engaged with discussion forums or direct peer messaging in the first 30 days, with a median number of engagements across the cohort of 0 (IQR 0-0). Significant differences were observed across providers for *Peer Support* engagement (N=12,857; χ^2^_2_=4337, *P*<.001). Provider D participants had a median of 2 (IQR 0-12) peer engagements, providers A and C participants had a median of 0 (IQR 0-0) peer engagements. More participants from provider D had a record of engaging in *Peer Support* features than participants from the other providers (1166/1944, 59.98% compared with 478/9821, 4.87% and 118/1087, 10.86% for providers A and C, respectively). This provider was also the only one to offer direct peer messaging (such as WhatsApp), whereas the other 2 providers offered a discussion forum (such as Facebook).

## Discussion

### Principal Findings

This study sought to explore engagement with the NHS-digital-DPP, looking at the way in which providers of this program promote participant engagement and exploring participants’ early engagement with the program apps.

The key findings, drawing together the quantitative and qualitative data, are now discussed.

Overall, for participants who had enrolled in the apps, engagement was high, with almost all participants (12,133/12,857, 94.37%) having used the apps to some degree. As highlighted in the provider interviews, there was a range of engagement, with some participants never engaging and some engaging every day. Providers had set expectations that their programs should be engaged with on a daily basis and used techniques such as prompts and reminders to encourage participants to do so. However, the use data suggest that this may not have been a realistic expectation as, on average, people engaged for 11 of the 30 days with the apps, with only 11.77% (1513/12,857) engaging every day. On average, participants spent a median of 60 (IQR 4-204) minutes using the apps; although, again, the ranges in the data showed that there were people who spent considerably more time than this.

Overall, tracking features were the most commonly engaged with app features, used by 83.91% (10,788/12,857) of participants. On average, participants engaged with the tracking features a median of 46 (IQR 3-22) times. Providers discussed behavior change techniques, including goal setting and self-monitoring behaviors, as important for engagement and operationalized these techniques through the goal setting and tracking features.

In contrast, the least engaged with features were the peer support features, which were minimally used by participants across all providers, with only 13.71% (1763/12,857) of participants engaging with peer support (a median of 0, IQR 0-0 interactions). Qualitative data highlighted that providers were aware of difficulties engaging participants with these features and discussed strategies they had tried to promote their use, such as trying to establish groups of users based on shared characteristics such as age. Providers also discussed the fact that peer support would really appeal to some participants and not others, and this is reflected in the range of engagement, with some people not engaging at all with these features and some logging up to 186 engagements with peer support features in the 30 days. Participants from provider D engaged more with the peer support features than participants from the other 2 providers, which may be a reflection of the availability of closed group chats in this app compared with those of the other 2 providers, who offered peer support in the form of open group discussion forums.

There were significant differences across providers in the amount and depth of engagement with the apps. Provider D had the most recorded use (time and interactions) over the 30 days and significantly more engagement with *Learn* features than the other 2 providers. The qualitative findings offer a potential explanation for this as this provider made new content available to participants on a daily basis, unlike the other 2 providers, who either released content every 30 days or tailored the arrival of new content based on how much participants were engaging.

Provider C recorded the highest number of days of use compared with the other 2 providers, with participants engaging for a median of 23 (IQR 12-29) days out of the 30. Of note, all participants from this provider had engaged at least once with a health coach during this period (average of 20). One of the main strategies that providers discussed to promote frequent engagement was interactions with health coaches, which this provider used as an opportunity to set expectations about participants’ frequency of use. This provider also had onboarding calls in which service users and health coaches made commitments to one another about engaging with the apps.

### Comparison With Previous Literature

Overall rates of engagement with the apps in this study appear to be high. Comparison with other studies of DHI use is made difficult by the multiple measures of engagement used throughout the literature and the different study designs. A randomized controlled trial of an internet-based diabetes prevention intervention reported that 99.5% of participants logged in to the intervention at least once during the first 6 months [48], and a process evaluation of a diabetes and prediabetes digital intervention showed that 74% of participants engaged once or more during a 16-week program [58]. This compares with 94.37% (12,133/12,857) of participants in this study having a record of some engagement with the apps in the first 30 days. The high rates of engagement in this study are also noteworthy given that these data are routinely collected and that adherence to DHIs in observational studies compared with randomized controlled trials has been found to be significantly lower [59,60].

DHI design features are not always well described, limiting the understanding of what the important features might be and why. This study explored the ways in which providers developed the NHS-digital-DPP interventions to promote participant engagement with them. Findings from the mixed methods analysis suggest that of particular importance for early engagement are features that allow participants to set goals and track behaviors and outcomes (tracking features) and interactions with health coaches. These findings complement other studies from this team that have focused on participant experiences of using the apps, which have found that tracking features and health coaches were beneficial in promoting participants’ subjective experiences of engagement [28] and that health coaches helped increase participants’ understanding of some features of the apps, such as setting goals, whereas participants reported being able to make sense of and use tracking features without support [61]. This may help explain why health coaches appear to be crucial for overall engagement as they help facilitate participants making use of other features, and the frequency of interactions with professionals (such as health coaches) has been found to be a significant predictor of adherence and engagement with DHIs in other studies [62,63]. This also explains why tracking was so high across providers as participants were able to use these features with no support.

In relation to our suggestion that the daily release of new content might be important for engagement, previous studies have also noted that novelty*,* generated by regular content updates, has been found to positively influence engagement with DHIs by preventing boredom [59,62] and that reminders, reported as an engagement strategy by all 4 providers in this study, are frequently reported to promote engagement with DHIs [32,64]. It has been suggested that hybrid systems that combine automated regular app content with elements of human support may achieve higher rates of engagement [32].

Peer support features were seldom used. Peer support may provide the opportunity to connect with others facing similar diagnoses and can provide quick, easy, and often anonymous access to emotional support, information based on others’ personal experiences, and resources that individuals may need to manage their illnesses [65]. An emerging body of literature suggests that peer support is important for engagement with digital diabetes prevention and outcomes [66,67]. The pilot study of the NHS-digital-DPP found that participants who had access to peer support lost more weight at 12 months than participants using interventions that did not have these features [17]. However, qualitative findings from NHS-digital-DPP participants suggested that there were mixed views on using peer support. Many reported not wanting or needing to engage with peers, and those who did reported that the peer support features were underused by other users, decreasing their motivation to engage with these aspects [28]. It may be that the findings of this study are influenced by the focus on early use. It has been suggested that the key to successful peer support forums is a critical mass of active users [68] who help the community thrive and self-regulate and a select minority of users—the superusers—who keep the community engaged and cohesive [65]. However, a longitudinal analysis of NHS-digital-DPP use data also shows the underuse of peer support features [40]. The findings are also in line with studies that suggest that the type of peer support offered may be of importance (Cheung, WC, unpublished data, 2023) as the participants who had access to closed group messaging (provider D) engaged more with peer support features than those who only had access to discussion forum features (provider C). Future work could look into differences in engagement with different types of peer support features.

### Strengths and Limitations

This study has several strengths. This is the first analysis of the early use of a nationally implemented digital health service anywhere in the world, and the findings reflect an independent analysis of routinely collected use data. In addition, this is one of the largest samples of any DHI use data study, representing >12,500 participants. The analysis of use data from 3 different providers was a further strength of this study. The mixed methods approach to this study allows for a better understanding of the early use of the NHS-digital-DPP, with qualitative findings providing explanations of and context to the quantitative findings. Individual-level data on referrals from the DPP minimum data set contain information on all referrals received by the DPP providers (Multimedia Appendix 2). Although comparisons are hampered by missing data, the overall impression is that providers managed participants with broadly similar age, sex, and deprivation profiles, suggesting that differences in early use are likely attributable to the different programs, at least in part. Therefore, this study’s findings on the features that may promote engagement may be generalizable to other DHIs.

In terms of limitations, despite substantial efforts, we were unable to obtain use data from one of the digital providers, and the data obtained from providers differed according to provider reporting capabilities and specific program features. For example, regarding measures of coach interactions, we compared frequency via apps, but we were unable to say anything about the quality of these interactions. Provider A, for example, recorded the fewest coach engagements but offered monthly calls with coaches (that were not captured in these analyses) compared with other providers that had a higher frequency of engagement but via messaging (which was captured in these analyses). Recent work has suggested that the intensity of such support from health coaches could be important [39]. There were also several limitations to the way in which the providers collected their data. First, the data we were provided with only captured use of the provider *apps*; for some of the providers, there were other platforms (such as websites) or routes of engagement with the program (eg, telephone calls) for participants that were not included in these analyses. Unfortunately, we were not able to assess the magnitude of this issue as data on this were not available from providers who did not record this. We were also informed by provider A that there was the possibility that engagement with the apps that was very quick was not logged by the data collection systems. Thus, our findings may not constitute a full representation of all engagement with the programs.

The qualitative analysis presents a set of factors that providers describe as being designed to promote participant engagement with their apps. As the original interviews were not informed by the DHI engagement framework, it is hard to tell whether the factors that were not described reflect evidence of absence or absence of evidence. However, interviews did probe respondents about the specific ways in which providers tried to engage participants; therefore, it is likely that all the most prominent features were discussed.

### Conclusions

This study presents an analysis of how the apps that comprise the NHS-digital-DPP have been designed to promote participant engagement. A key strength is the analysis of real-world data on early engagement with the NHS-digital-DPP apps for participants enrolled during a 9-month period, presenting one of the few examples of an analysis of routinely collected use data of this population. Overall, engagement with the NHS-digital-DPP apps was high, with a large proportion of participants engaging and high rates of use of several key intervention components. However, variability was observed across providers, and a key limitation was the absence of data from one provider. Implications for developers of digital services such as the NHS-digital-DPP include the importance of self-monitoring and health coaches for engagement. Future research could extend this work by exploring how users from different demographic groups engage with the NHS-digital-DPP and whether specific program features result in better outcomes.

## Appendix

Multimedia Appendix 1 Conceptual framework of direct and indirect influences on engagement with digital behavior change interventions.

Multimedia Appendix 2 Data from the minimum data set on the characteristics of participants referred to the National Health Service Digital Diabetes Prevention Programme.

## Abbreviations

DHI: digital health intervention
DPP: diabetes prevention program
NHS: National Health Service
NHS-digital-DPP: Healthier You National Health Service Digital Diabetes Prevention Programme
RQ: research question
T2DM: type 2 diabetes mellitus

## Data Availability

The materials from digital providers and audio recordings of interviews analyzed in this study are not publicly available because of confidentiality agreements with the provider organizations as some information is commercially sensitive. Some data sets may be available from the corresponding author upon reasonable request, although the authors will require explicit permission from the relevant provider organizations.

## References

[1] (2023). Diabetes. World Health Organization.

[2] Diabetes prevalence 2019. Diabetes UK.

[3] (2015). NHS Diabetes Prevention Programme (NHS DPP) non-diabetic hyperglycaemia. Public Health England.

[4] Lindström J, Ilanne-Parikka P, Peltonen M, Aunola S, Eriksson JG, Hemiö K, Hämäläinen H, Härkönen P, Keinänen-Kiukaanniemi S, Laakso M, Louheranta A, Mannelin M, Paturi M, Sundvall J, Valle TT, Uusitupa M, Tuomilehto J (2006). Sustained reduction in the incidence of type 2 diabetes by lifestyle intervention: follow-up of the Finnish Diabetes Prevention Study. Lancet.

[5] Uusitupa M, Khan TA, Viguiliouk E, Kahleova H, Rivellese AA, Hermansen K, Pfeiffer A, Thanopoulou A, Salas-Salvadó J, Schwab U, Sievenpiper JL (2019). Prevention of type 2 diabetes by lifestyle changes: a systematic review and meta-analysis. Nutrients.

[6] Galaviz KI, Weber MB, Straus A, Haw JS, Narayan KM, Ali MK (2018). Global diabetes prevention interventions: a systematic review and network meta-analysis of the real-world impact on incidence, weight, and glucose. Diabetes Care.

[7] Dunkley AJ, Bodicoat DH, Greaves CJ, Russell C, Yates T, Davies MJ, Khunti K (2014). Diabetes prevention in the real world: effectiveness of pragmatic lifestyle interventions for the prevention of type 2 diabetes and of the impact of adherence to guideline recommendations: a systematic review and meta-analysis. Diabetes Care.

[8] (2016). NHS Diabetes Prevention Programme national service specification. National Health Service England.

[9] Valabhji J, Barron E, Bradley D, Bakhai C, Fagg J, O'Neill S, Young B, Wareham N, Khunti K, Jebb S, Smith J (2020). Early outcomes from the English National Health Service Diabetes Prevention Programme. Diabetes Care.

[10] McManus E, Meacock R, Parkinson B, Sutton M (2022). Population level impact of the NHS Diabetes Prevention Programme on incidence of type 2 diabetes in England: an observational study. Lancet Reg Health Eur.

[11] Marsden AM, Bower P, Howarth E, Soiland-Reyes C, Sutton M, Cotterill S (2022). 'Finishing the race' - a cohort study of weight and blood glucose change among the first 36,000 patients in a large-scale diabetes prevention programme. Int J Behav Nutr Phys Act.

[12] Halley MC, Petersen J, Nasrallah C, Szwerinski N, Romanelli R, Azar KM (2020). Barriers and facilitators to real-world implementation of the diabetes prevention program in large healthcare systems: lifestyle coach perspectives. J Gen Intern Med.

[13] Harrison CR, Phimphasone-Brady P, DiOrio B, Raghuanath SG, Bright R, Ritchie ND, Sauder KA (2020). Barriers and facilitators of National Diabetes Prevention Program engagement among women of childbearing age: a qualitative study. Diabetes Educ.

[14] McGough B (2018). Going digital to expand the Diabetes Prevention Programme. Pract Nurs.

[15] Rose T, Barker M, Maria Jacob C, Morrison L, Lawrence W, Strömmer S, Vogel C, Woods-Townsend K, Farrell D, Inskip H, Baird J (2017). A systematic review of digital interventions for improving the diet and physical activity behaviors of adolescents. J Adolesc Health.

[16] Beleigoli AM, Andrade AQ, Cançado AG, Paulo MN, Diniz MD, Ribeiro AL (2019). Web-based digital health interventions for weight loss and lifestyle habit changes in overweight and obese adults: systematic review and meta-analysis. J Med Internet Res.

[17] Ross JA, Barron E, McGough B, Valabhji J, Daff K, Irwin J, Henley WE, Murray E (2022). Uptake and impact of the English National Health Service digital diabetes prevention programme: observational study. BMJ Open Diabetes Res Care.

[18] Sepah SC, Jiang L, Ellis RJ, McDermott K, Peters AL (2017). Engagement and outcomes in a digital Diabetes Prevention Program: 3-year update. BMJ Open Diabetes Res Care.

[19] Joiner KL, Nam S, Whittemore R (2017). Lifestyle interventions based on the diabetes prevention program delivered via eHealth: a systematic review and meta-analysis. Prev Med.

[20] Van Rhoon L, Byrne M, Morrissey E, Murphy J, McSharry J (2020). A systematic review of the behaviour change techniques and digital features in technology-driven type 2 diabetes prevention interventions. Digit Health.

[21] Marsden AM, Hann M, Barron E, Ross J, Valabhji J, Murray E, Cotterill S (2023). Comparison of weight change between face-to-face and digital delivery of the English National Health service diabetes prevention programme: an exploratory non-inferiority study with imputation of plausible weight outcomes. Prev Med Rep.

[22] Barron E, Bradley D, Safazadeh S, McGough B, Bakhai C, Young B, Khunti K, Murray E, Wareham N, Jebb S, Valabhji J (2023). Effectiveness of digital and remote provision of the Healthier You: NHS Diabetes Prevention Programme during the COVID-19 pandemic. Diabet Med.

[23] Grock S, Ku J-H, Kim J, Moin T (2017). A review of technology-assisted interventions for diabetes prevention. Curr Diab Rep.

[24] Sweet CC, Jasik CB, Diebold A, DuPuis A, Jendretzke B (2020). Cost savings and reduced health care utilization associated with participation in a digital diabetes prevention program in an adult workforce population. J Health Econ Outcomes Res.

[25] Murray E, Burns J, May C, Finch T, O'Donnell C, Wallace P, Mair F (2011). Why is it difficult to implement e-health initiatives? A qualitative study. Implement Sci.

[26] Ross J, Stevenson F, Lau R, Murray E (2016). Factors that influence the implementation of e-health: a systematic review of systematic reviews (an update). Implement Sci.

[27] Estacio EV, Whittle R, Protheroe J (2019). The digital divide: examining socio-demographic factors associated with health literacy, access and use of internet to seek health information. J Health Psychol.

[28] Ross J, Cotterill S, Bower P, Murray E (2023). Influences on patient uptake of and engagement with the National Health Service Digital Diabetes Prevention Programme: qualitative interview study. J Med Internet Res.

[29] Cole-Lewis H, Ezeanochie N, Turgiss J (2019). Understanding health behavior technology engagement: pathway to measuring digital behavior change interventions. JMIR Form Res.

[30] Perski O, Blandford A, West R, Michie S (2017). Conceptualising engagement with digital behaviour change interventions: a systematic review using principles from critical interpretive synthesis. Transl Behav Med.

[31] Canhoto AI, Arp S (2016). Exploring the factors that support adoption and sustained use of health and fitness wearables. J Mark Manag.

[32] Jakob R, Harperink S, Rudolf AM, Fleisch E, Haug S, Mair JL, Salamanca-Sanabria A, Kowatsch T (2022). Factors influencing adherence to mHealth apps for prevention or management of noncommunicable diseases: systematic review. J Med Internet Res.

[33] Wei Y, Zheng P, Deng H, Wang X, Li X, Fu H (2020). Design features for improving mobile health intervention user engagement: systematic review and thematic analysis. J Med Internet Res.

[34] Morrison LG, Yardley L, Powell J, Michie S (2012). What design features are used in effective e-health interventions? A review using techniques from Critical Interpretive Synthesis. Telemed J E Health.

[35] Michaelides A, Raby C, Wood M, Farr K, Toro-Ramos T (2016). Weight loss efficacy of a novel mobile Diabetes Prevention Program delivery platform with human coaching. BMJ Open Diabetes Res Care.

[36] Lavikainen P, Mattila E, Absetz P, Harjumaa M, Lindström J, Järvelä-Reijonen E, Aittola K, Männikkö R, Tilles-Tirkkonen T, Lintu N, Lakka T, van Gils M, Pihlajamäki J, Martikainen J (2022). Digitally supported lifestyle intervention to prevent type 2 diabetes through healthy habits: secondary analysis of long-term user engagement trajectories in a randomized controlled trial. J Med Internet Res.

[37] Hori JH, Sia EX, Lockwood KG, Auster-Gussman LA, Rapoport S, Branch OH, Graham SA (2022). Discovering engagement personas in a digital diabetes prevention program. Behav Sci (Basel).

[38] Hawkes RE, Miles LM, French DP (2023). What behaviour change technique content is offered to service users of the nationally implemented English NHS Digital Diabetes Prevention Programme: analysis of multiple sources of intervention content. Prev Med Rep.

[39] Miles LM, Hawkes RE, French DP (2023). Description of the nationally implemented National Health Service digital diabetes prevention programme and rationale for its development: mixed methods study. BMC Health Serv Res.

[40] Hawkes RE, Miles LM, Ainsworth B, Ross J, Meacock R, French DP (2023). Engagement with a nationally-implemented digital behaviour change intervention: usage patterns over the 9-month duration of the National Health Service Digital Diabetes Prevention Programme. Internet Interv.

[41] Dennison L, Morrison L, Conway G, Yardley L (2013). Opportunities and challenges for smartphone applications in supporting health behavior change: qualitative study. J Med Internet Res.

[42] Tomlinson M, Rotheram-Borus MJ, Swartz L, Tsai AC (2013). Scaling up mHealth: where is the evidence?. PLoS Med.

[43] Struik LL, Bottorff JL, Baskerville NB, Oliffe J, Crichton S (2019). Comparison of developers' and end-users' perspectives about smoking cessation support through the crush the crave app. JMIR Mhealth Uhealth.

[44] Baumel A, Fleming T, Schueller SM (2020). Digital micro interventions for behavioral and mental health gains: core components and conceptualization of digital micro intervention care. J Med Internet Res.

[45] Baumel A, Edan S, Kane JM (2019). Is there a trial bias impacting user engagement with unguided e-mental health interventions? A systematic comparison of published reports and real-world usage of the same programs. Transl Behav Med.

[46] Yardley L, Spring BJ, Riper H, Morrison LG, Crane DH, Curtis K, Merchant GC, Naughton F, Blandford A (2016). Understanding and promoting effective engagement with digital behavior change interventions. Am J Prev Med.

[47] O'Brien HL, Toms EG (2008). What is user engagement? A conceptual framework for defining user engagement with technology. J Am Soc Inf Sci Technol.

[48] Harjumaa M, Absetz P, Ermes M, Mattila E, Männikkö R, Tilles-Tirkkonen T, Lintu N, Schwab U, Umer A, Leppänen J, Pihlajamäki J (2020). Internet-based lifestyle intervention to prevent type 2 diabetes through healthy habits: design and 6-month usage results of randomized controlled trial. JMIR Diabetes.

[49] Truby H, Baic S, deLooy A, Fox KR, Livingstone MB, Logan CM, Macdonald IA, Morgan LM, Taylor MA, Millward DJ (2006). Randomised controlled trial of four commercial weight loss programmes in the UK: initial findings from the BBC "diet trials". BMJ.

[50] (2019). Service specification. National Health Service.

[51] Hanson WE, Creswell JW, Clark VL, Petska KS, Creswell JD (2005). Mixed methods research designs in counseling psychology. J Counsel Psychol.

[52] Johnson RB, Onwuegbuzie AJ, Turner LA (2016). Toward a definition of mixed methods research. J Mixed Methods Res.

[53] Type 2 diabetes know your risk. Diabetes UK.

[54] Hawkes RE, Miles LM, French DP (2022). Fidelity to program specification of the national health service digital diabetes prevention program behavior change technique content and underpinning theory: document analysis. J Med Internet Res.

[55] (2017). NVivo (Version 12). Lumivero.

[56] Michie S, Richardson M, Johnston M, Abraham C, Francis J, Hardeman W, Eccles MP, Cane J, Wood CE (2013). The behavior change technique taxonomy (v1) of 93 hierarchically clustered techniques: building an international consensus for the reporting of behavior change interventions. Ann Behav Med.

[57] (2017). IBM SPSS Statistics for Windows, Version 25.0. IBM Corp.

[58] Signal V, McLeod M, Stanley J, Stairmand J, Sukumaran N, Thompson D-M, Henderson K, Davies C, Krebs J, Dowell A, Grainger R, Sarfati D (2020). A mobile- and web-based health intervention program for diabetes and prediabetes self-management (BetaMe/Melon): process evaluation following a randomized controlled trial. J Med Internet Res.

[59] Kelders SM, Kok RN, Ossebaard HC, Van Gemert-Pijnen JE (2012). Persuasive system design does matter: a systematic review of adherence to web-based interventions. J Med Internet Res.

[60] Christensen H, Griffiths KM, Korten AE, Brittliffe K, Groves C (2004). A comparison of changes in anxiety and depression symptoms of spontaneous users and trial participants of a cognitive behavior therapy website. J Med Internet Res.

[61] Miles LM, Hawkes RE, French DP (2023). How the behavior change content of a nationally implemented digital diabetes prevention program is understood and used by participants: qualitative study of fidelity of receipt and enactment. J Med Internet Res.

[62] Brouwer W, Kroeze W, Crutzen R, de Nooijer J, de Vries NK, Brug J, Oenema A (2011). Which intervention characteristics are related to more exposure to internet-delivered healthy lifestyle promotion interventions? A systematic review. J Med Internet Res.

[63] Mohr DC, Cuijpers P, Lehman K (2011). Supportive accountability: a model for providing human support to enhance adherence to eHealth interventions. J Med Internet Res.

[64] Alkhaldi G, Modrow K, Hamilton F, Pal K, Ross J, Murray E (2017). Promoting engagement with a digital health intervention (HeLP-Diabetes) using email and text message prompts: mixed-methods study. Interact J Med Res.

[65] De Simoni A, Taylor SJ, Griffiths C, Panzarasa P, Sheikh A (2018). Online “superusers” as allies of the health care workforce. NEJM Catalyst.

[66] Ekezie W, Dallosso H, Saravanan P, Khunti K, Hadjiconstantinou M (2021). Experiences of using a digital type 2 diabetes prevention application designed to support women with previous gestational diabetes. BMC Health Serv Res.

[67] Skoglund G, Nilsson BB, Olsen CF, Bergland A, Hilde G (2022). Facilitators and barriers for lifestyle change in people with prediabetes: a meta-synthesis of qualitative studies. BMC Public Health.

[68] Kim H-S, Sundar SS (2016). Motivating contributions to online forums: can locus of control moderate the effects of interface cues?. Health Commun.

