# Supplementary material for: Design and Early Use of the Nationally Implemented Healthier You National Health Service Digital Diabetes Prevention Programme: Mixed Methods Study
Source: J Med Internet Res. 2023 Aug 17;25:e47436. doi: 10.2196/47436 (PMC10472174; doi:10.2196/47436)
Supplement: Multimedia Appendix 1 [file jmir_v25i1e47436_app1.docx]

Multimedia appendix 1

Conceptual framework of direct and indirect influences on engagement with DBCIs. Data were coded to the concepts in the blue boxes on the left that relate to intervention content and delivery.


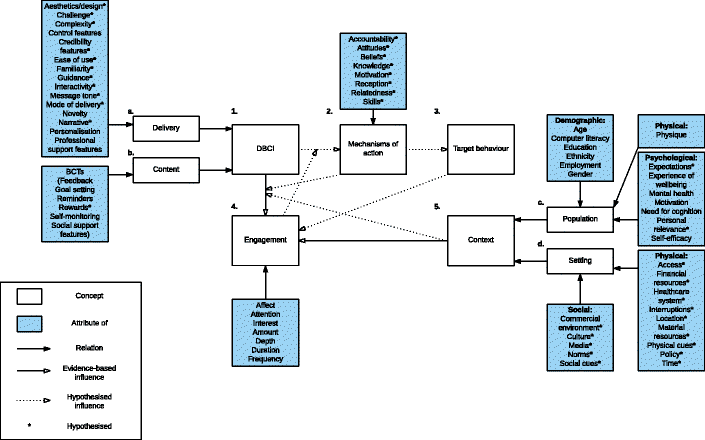


Figure shared from Perski, O., Blandford, A., West, R. et al. Conceptualising engagement with digital behaviour change interventions: a systematic review using principles from critical interpretive synthesis. Behav. Med. Pract. Policy Res. 7, 254–267 (2017). <https://doi.org/10.1007/s13142-016-0453-1> under the terms of Creative Commons Attribution 4.0 International License (<https://creativecommons.org/licenses/by/4.0/>) No changes have been made.
