# Supplementary material for: Design and Early Use of the Nationally Implemented Healthier You National Health Service Digital Diabetes Prevention Programme: Mixed Methods Study
Source: J Med Internet Res. 2023 Aug 17;25:e47436. doi: 10.2196/47436 (PMC10472174; doi:10.2196/47436)
Supplement: Multimedia Appendix 2 [file jmir_v25i1e47436_app2.docx]

Multimedia appendix 2

Data from the Minimum data set (MDS) ^a^ on characteristics of participants referred to the NHS-digital-DPP during the study period (between 1st December 2020 and 28th February 2022)

| Demographic data of participants referred on the Digital NHS-DPP | | | | | | | |
| --- | --- | --- | --- | --- | --- | --- | --- |
|  | All | Provider A | Provider A^c^ | Provider B | Provider C | Provider D |  |
| Number referred | n=74564^b^ | n=47735 | n=11275 | n=7714 | n=2675 | n=5165 |  |
| Male (%) | 44.1% | 45.7% | 39.8% | 41.8% | 47.0% | 41.5% |  |
| Age at referral  Mean (SD) | 59.7 (14.0) | 61.1 (13.9) | 57.9 (14.5) | 58.9 (13.8) | 54.9 (12.3) | 54.1 (12.9) |  |
| IMD Quintile |  |  |  |  |  |  |  |
| 1 | 20.4% | 17.6% | 31.8% | 19.5% | 18.5% | 23.4% |  |
| 2 | 20.7% | 21.1% | 20.1% | 19.8% | 19.2% | 20.9% |  |
| 3 | 20.1% | 20.7% | 16.6% | 19.9% | 22.8% | 21.3% |  |
| 4 | 19.7% | 20.7% | 15.6% | 20.1% | 19.9% | 18.9% |  |
| 5 | 19.0% | 19.8% | 15.8% | 20.5% | 19.4% | 15.1% |  |
| Missing | 0.1% | 0.0% | 0.0% | 0.2% | 0.3% | 0.4% |  |
| Ethnicity |  |  |  |  |  |  |  |
| White | 36.8% | 19.1% | 50.1% | 80.6% | 80.9% | 83.1% |  |
| Asian | 6.6% | 5.4% | 9.1% | 7.9% | 8.4% | 8.8% |  |
| Black | 2.8% | 2.6% | 3.4% | 1.9% | 3.7% | 4.5% |  |
| Mixed | 2.1% | 1.9% | 1.7% | 2.7% | 4.8% | 2.9% |  |
| Missing | 51.6% | 70.9% | 35.6% | 6.8% | 2.2% | 0.7% |  |
| BMI Category |  |  |  |  |  |  |  |
| Underweight / healthy | 5.1% | 2.7% | 6.1% | 11.7% | 12.0% | 11.6% |  |
| Overweight | 10.3% | 4.9% | 12.4% | 23.7% | 27.5% | 25.7% |  |
| Obese | 19.4% | 7.1% | 31.4% | 41.8% | 46.6% | 59.5% |  |
| Missing | 65.2% | 85.3% | 50.0% | 22.8% | 13.9% | 3.2% |  |

^a^ The Minimum Data Set (MDS) contains information about all referrals made to the NHS DPP, collected by programme providers. This dataset includes the date a referral was made and the subsequent level of attendance of that individual on the programme. As part of the referral, basic demographic information was sent to providers including sex, age and index of multiple deprivation. For individuals who then attended the initial assessment, further information was collected including the individual’s ethnicity, weight, body mass index (BMI) and health-related quality of life (measured using the EQ-5D-5L).

^b^ These figures are larger than the data presented in the paper as this data reflects all participants who were referred during the entire 15 month study period, whereas data in the paper reflects 9 months of data for each provider. This data also included MDS data for provider B.

^c^ The digital providers of the NHS-DPP are subcontracted by 5 face-to-face providers of the NHS-DPP. Provider A was subcontracted by two of the face-to-face providers, and therefore has two entries.
